# Rivaroxaban Plus Aspirin for Extended Thromboprophylaxis in Acutely Ill Medical Patients: Insights from the MARINER Trial

**DOI:** 10.1055/s-0042-1750379

**Published:** 2022-07-11

**Authors:** Alex C. Spyropoulos, Mark Goldin, Walter Ageno, Gregory W. Albers, C. Gregory Elliott, William R. Hiatt, Jonathan L. Halperin, Gregory Maynard, P. Gabriel Steg, Jeffrey I. Weitz, Theodore E. Spiro, Wentao Lu, Jessica Marsigliano, Gary E. Raskob, Elliot S. Barnathan

**Affiliations:** 1 The Donald and Barbara Zucker School of Medicine at Hofstra/Northwell, The Feinstein Institute for Medical Research, New York, New York, United States; 2 Department of Medicine, Anticoagulation and Clinical Thrombosis Services, Northwell Health at Lenox Hill Hospital, New York, New York, United States; 3 Northwell Health, Great Neck, New York, United States; 4 Department of Medicine and Surgery, University of Insubria, Varese, Italy; 5 University of Insubria, Varese, Italy; 6 Stanford Stroke Center, Stanford Medical Center, Stanford University, Palo Alto, California, United States; 7 Department of Medicine, University of Utah and Intermountain Healthcare, Salt Lake City, Utah, United States; 8 Division of Cardiology, University of Colorado School of Medicine; 9 CPC Clinical Research, Aurora, Colorado, United States; 10 Cardiovascular Institute, Mount Sinai Medical Center, New York, New York, United States; 11 University of California at Davis, Sacramento, California, United States; 12 Université de Paris, Assistance Publique–Hôpitaux de Paris, and INSERM U-1148, Paris, France; 13 McMaster University; 14 The Thrombosis and Atherosclerosis Research Institute, Hamilton, Ontario, Canada; 15 Bayer US, LLC, Whippany, New Jersey, United States; 16 Cardiovascular Clinical Development, Janssen Research and Development, LLC, Raritan, New Jersey, United States; 17 Hudson College of Public Health, University of Oklahoma Health Sciences Center, Oklahoma City, Oklahoma, United States

**Keywords:** aspirin, combined modality therapy, hospitalization, rivaroxaban, venous thromboembolism

## Abstract

**Background** The MARINER trial evaluated whether postdischarge thromboprophylaxis with rivaroxaban could reduce the primary outcome of symptomatic venous thromboembolism (VTE) or VTE-related death in acutely ill medical patients at risk for VTE. Although aspirin use was not randomized, approximately half of the enrolled patients were receiving aspirin at baseline. We hypothesized that thromboprophylaxis with once-daily rivaroxaban (10 mg or, if creatinine clearance was 30–49 mL/min, 7.5 mg) plus aspirin (R/A) would be superior to placebo without aspirin (no thromboprophylaxis [no TP]).

**Methods** We compared the primary and major secondary outcomes in the intention-to-treat population in four subgroups defined at baseline: (1) R/A ( *N* = 3,159); (2) rivaroxaban alone ( *N* = 2,848); (3) aspirin alone ( *N* = 3,046); and (4) no TP ( *N* = 2,966). Major bleeding (MB) and nonmajor clinically relevant (NMCR) bleeding were assessed in the safety population on treatment plus 2 days.

**Results** Patients on R/A had reduced symptomatic VTE and VTE-related death compared with no TP (0.76 vs 1.28%, *p* = 0.042), and experienced less symptomatic VTE and all-cause mortality ( *p* = 0.005) and all-cause mortality alone ( *p* = 0.01) compared with no TP. Event incidences for rivaroxaban alone (0.91%) or aspirin alone (0.92%) were similar. MB was low in all groups but lowest in the no TP group. NMCR bleeding was increased with R/A compared with no TP ( *p* = 0.009).

**Limitations** Aspirin use was not randomized.

**Conclusion** Extended postdischarge thromboprophylaxis with R/A was associated with less symptomatic VTE and VTE-related death compared with no TP in previously hospitalized medical patients at risk for VTE. NMCR bleeding was increased with R/A compared with no TP. These post hoc findings need confirmation in a prospective trial.

## Introduction

Patients hospitalized with acute medical illness are at increased risk for venous thromboembolism (VTE) during a hospital stay as well as after discharge. 1 2 While the benefits of inpatient anticoagulant thromboprophylaxis are well established, 3 postdischarge prophylaxis confers a trade-off between reduction in VTE and increased major bleeding (MB). 4 Professional society guidelines recommend thromboprophylaxis in hospitalized, medically ill patients with low-dose unfractionated heparin, low-molecular-weight heparin, or fondaparinux, but do not recommend routine use of postdischarge prophylaxis due to uncertain net clinical benefit. 5 6

The direct oral anticoagulants (DOACs) betrixaban and rivaroxaban have shown favorable benefit/risk profiles in trials of extended-duration postdischarge thromboprophylaxis for key medically ill patient subgroups at high risk of VTE and low bleed risk, 7 8 and have been approved by the U.S. Food and Drug Administration to reduce the risk of symptomatic VTE and VTE-related death following hospitalization for an acute medical illness. Low-dose aspirin may be similarly beneficial as postdischarge thromboprophylaxis, in particular, after hip or knee arthroplasty, and has been suggested for use by guidelines. 5 6 9 10 In addition, data suggest that dual pathway inhibition with low-dose anticoagulants plus aspirin is more effective than an anticoagulant alone in reducing major and fatal thromboembolic disease. 11 12 13 As such, the addition of aspirin to low-dose rivaroxaban (10 mg once daily) in medically ill patients has the potential to confer additional benefit. 14

The MARINER trial evaluated whether rivaroxaban given postdischarge reduced symptomatic VTE and VTE-related death among high-risk patients hospitalized with acute medical illness. 8 Rivaroxaban at the doses tested did not achieve a significant reduction in the primary end point of symptomatic VTE or VTE-related death, but did reduce the secondary end point of symptomatic VTE and all-cause mortality (ACM). 8 Although aspirin use was not randomized, more than half of the 12,019 patients in the MARINER trial were prescribed aspirin at baseline. It was therefore of interest to explore whether patients receiving dual pathway inhibition with rivaroxaban and aspirin might be associated with increased benefit compared with those receiving neither agent. For this post hoc analysis, we hypothesized that treatment with rivaroxaban plus aspirin would be superior to use of neither rivaroxaban nor aspirin in preventing major thromboembolic outcomes and death.

## Methods

The MARINER trial (NCT02111564) was a multicenter, randomized, double-blind, placebo-controlled, event-driven efficacy and safety study that evaluated rivaroxaban compared with placebo, in the prevention of symptomatic VTE events and VTE-related deaths post–hospital discharge in high-risk medically ill patients. MARINER was conducted at 671 centers in 36 countries from June 2014 through January 2018. Patients were randomized in a 1:1 ratio. Patients with normal renal function consisting of creatinine clearance ≥ 50 mL/min received rivaroxaban 10 mg once daily (or placebo), while those with renal impairment consisting of creatinine clearance 30 to 49 mL/min received rivaroxaban 7.5 mg once daily (or placebo). Treatments were given for a period of 45 days post–hospital discharge. While aspirin use was not randomized, approximately half of the enrolled patients were receiving low-dose aspirin (≤162 mg/day) at baseline, while the rest were not on any aspirin.

To be eligible, patients had to be at least 40 years of age and hospitalized for an acute medical condition such as heart failure, acute respiratory insufficiency or acute exacerbation of chronic obstructive pulmonary disease, acute ischemic stroke, acute infectious disease, or inflammatory disease including rheumatic disease for 3 to 10 consecutive days prior to randomization. Patients also needed to be at increased risk of VTE as demonstrated by an International Medical Prevention Registry on VTE (IMPROVE) risk score of 4 or greater (or a risk score of 2 or 3 with plasma D-dimer level ≥ 2× the upper limit of normal during the index hospitalization). Patients with active cancer, active gastrointestinal ulcer or significant bleeding within 3 months, history of hemorrhagic stroke, severe renal insufficiency or liver disease, or bronchiectasis, those who required anticoagulation, strong CYP3A4 inhibitors or inducers, aspirin >162 mg/day, clopidogrel >75 mg/day, or ticlopidine >250 mg twice daily, clopidogrel at any dose in combination with omeprazole or esomeprazole, dipyridamole >400 mg/day, cilostazol >200 mg/day, other P2Y12 receptor antagonists, or thrombin-receptor antagonists, or those who required dual antiplatelet therapy were excluded from the MARINER trial.

The primary hypothesis of the MARINER trial was that rivaroxaban was superior to placebo for the prevention of the composite outcome of symptomatic VTE (i.e., lower extremity deep vein thrombosis [DVT] or nonfatal pulmonary embolism [PE]) and VTE-related death (i.e., death due to PE or death in which PE could not be ruled out) and was assessed in the intention-to-treat (ITT) population. The results of this trial have been reported previously. 8 The hypothesis for this study was that dual antithrombotic therapy (rivaroxaban and low-dose aspirin) would be superior to no antithrombotic therapy.

Secondary efficacy end points assessed in the ITT population included: symptomatic VTE; VTE-related death; symptomatic VTE and ACM; symptomatic VTE/myocardial infarction (MI)/nonhemorrhagic stroke/cardiovascular (CV) death; and ACM. MB and nonmajor clinically relevant (NMCR) bleeding were assessed in the safety population from randomization to 2 days after the last dose of study medication. All end points were adjudicated by a central independent clinical events committee, which was blinded to treatment assignment (Appendix A).

Patients were categorized into one of four subgroups defined at baseline: (1) rivaroxaban alone (10 mg once daily for 45 days with dose reduction to 7.5 mg once daily if baseline creatinine clearance was 30–49 mL/min); (2) aspirin (≤162 mg daily) alone; (3) rivaroxaban plus aspirin (R/A); (4) neither rivaroxaban nor aspirin (no thromboprophylaxis [no TP]). Incidences were compared using chi-square testing, without adjustment for multiplicity. Aspirin use at baseline was not randomized and aspirin use beyond baseline was not investigated, although the protocol allowed aspirin use to continue post–hospital discharge at the discretion of the investigator.

As aspirin use at baseline was not randomized, we also performed a sensitivity analysis, where baseline demographic covariates (shown in Table 1 ) were tested for significant association with the primary end point using a Cox model with backward variable selection procedure using a cutoff of *p* < 0.05 to stay in the model. Those covariates that stayed in the model were then adjusted for in the final Cox proportional hazards model. This approach was also done for the composite end point of symptomatic VTE and ACM as well as for ACM alone.

**Table 1:** Demographics and baseline characteristics in MARINER by treatment group (ITT population)

	Rivaroxaban alone ( *N* = 2,848)	ASA alone ( *N* = 3,046)	Rivaroxaban + ASA ( *N* = 3,159)	No rivaroxaban + no ASA ( *N* = 2,966)	*p-* Value
Male, %	49.3	54.8	54.6	50.0	<0.0001
Race, %					
White	95.8	97.1	96.7	96.1	0.10
Black	1.2	0.7	1.2	1.2	
Asian	0.1	0.1	0.2	0.2	
Other a	3.0	2.1	1.9	2.5	
Ethnicity, %					
Hispanic or Latino	11.1	6.4	5.8	9.7	<0.0001
Not Hispanic or Latino	88.6	93.3	94.0	90.0	
Unknown	0.4	0.3	0.2	0.4	
Age (y), mean	69.4	69.8	70.0	69.5	0.13
Weight (kg), mean	79.2	81.3	82.2	80.0	<0.0001
CrCl (mL/min), %					
30 to <50	17.0	20.9	19.4	15.6	<0.0001
50 to <80	36.9	39.7	41.2	38.7	
≥80	46.0	39.4	39.4	45.7	
Height (cm), mean	166.3	167.9	167.6	166.7	<0.0001
BMI (kg/m ^2^ ), %					
< 25	29.6	24.4	22.9	27.6	0.0002
< 25 to <35	56.3	63.2	61.7	57.8	
≥35	14.2	12.4	15.4	14.5	
Systolic blood pressure (mm Hg), mean	128	129	129	128	0.075
Diastolic blood pressure (mm Hg), mean	76	77	77	77	0.047
Pulse rate (beats/min), mean	77	73	73	77	<0.0001
Smoking history, %					
Never used	52.8	56.1	56.3	53.0	<0.0001
Current user	18.5	14.8	14.8	18.1	
Former user	28.7	29.1	29.0	28.9	
History of cancer, %	8.8	7.8	7.5	9.9	0.0033
D-dimer, %					
> 2× ULN	73.6	68.4	67.4	72.7	<0.0001
≤2× ULN	18.8	25.0	26.6	20.2	
Not done	7.6	6.6	6.0	7.1	
Duration of hospital stay (mean), d	6.7	6.8	6.7	6.6	0.057
Previous VTE, %	12.6	11.5	12.9	13.4	0.17
Lower limb paralysis or paresis, %	13.6	24.7	23.0	12.5	<0.0001
Reason for hospitalization, %					
Acute ischemic stroke	8.1	20.7	19.9	7.9	<0.0001
Acute infectious disease	24.3	10.4	11.3	24.6	
Inflammatory disease	2.2	0.5	0.7	2.5	
Acute respiratory insufficiency	37.5	16.9	16.0	37.0	
Baseline heart failure	27.9	51.5	51.9	28.0	
Modified IMPROVE score, %					
2	41.4	29.7	29.1	42.0	<0.0001
3	30.0	31.9	32.7	27.2	
≥4	28.5	38.4	38.2	30.4	

Abbreviations: ASA, aspirin; BMI, body mass index; CrCl, creatinine clearance; ITT, intention to treat; ULN, upper limit of normal; VTE, venous thromboembolism.

Note: Chi-square test and one-way analysis of variance test were used for categorical variables and continuous variables, respectively.

a Other includes “American Indian or Alaskan native,” “Native Hawaiian or Other Pacific Islander,” “Other,” “Multiple,” and “Unknown” on the case report form.

## Results

The baseline demographic and clinical characteristics of the patients in each subgroup are shown in Table 1 . Overall, in groups taking aspirin there were more males, fewer Hispanic or Latino patients, and fewer smokers and patient with a history of cancer, but more patients with renal insufficiency, lower limb paralysis or paresis, and hospitalization for acute ischemic stroke or heart failure.

The results of the primary and major secondary efficacy and safety end points by subgroup are displayed in Table 2 . R/A was associated with a significant reduction of the primary efficacy outcome of symptomatic VTE and VTE-related death (0.76 vs 1.28%, *p* = 0.042) compared with no TP. Additionally, the event incidences for rivaroxaban alone (0.91%) or aspirin alone (0.92%) were similar and numerically higher than with combination therapy (0.76%). The combination of rivaroxaban and aspirin was associated with a significant reduction of symptomatic VTE and ACM (1.20 vs 2.12%, *p* = 0.005), as well as ACM alone (1.04 vs 1.82%, *p* = 0.01), compared with no TP. Of note, the aspirin-alone group was also associated with reduced ACM (1.15 vs 1.82%, *p* = 0.03).

**Table 2:** Effectiveness and safety ed points in MARINER by treatment group

Effectiveness					
End point (up to day 45 in ITT population)	Rivaroxaban alone ( *N* = 2,848) *n* (%)	ASA alone ( *N* = 3,046) *n* (%)	Rivaroxaban + ASA ( *N* = 3,159) *n* (%)	No rivaroxaban + no ASA ( *N* = 2,966) *n* (%)	*p-* Value (rivaroxaban + ASA vs no rivaroxaban + no ASA)
Sx VTE + VTE-related death	26 (0.91)	28 (0.92)	24 (0.76)	38 (1.28)	0.042
VTE-related death	24 (0.84)	18 (0.59)	19 (0.60)	28 (0.94)	0.13
Sx VTE	5 (0.18)	13 (0.43)	6 (0.19)	12 (0.40)	0.12
Sx VTE and ACM	40 (1.40)	44 (1.44)	38 (1.20)	63 (2.12)	0.005
Sx VTE, MI, nonhemorrhagic stroke and CV death	44 (1.54)	57 (1.87)	50 (1.58)	63 (2.12)	0.12
ACM	38 (1.33)	35 (1.15)	33 (1.04)	54 (1.82)	0.010
**Safety**					
**End point (on treatment +2 days in safety population)**	**Rivaroxaban alone** **(** ***N*** ** = 2,833)** ***n*** **(%)**	**ASA alone** **(** ***N*** ** = 3,032)** ***n*** **(%)**	**Rivaroxaban + ASA** **(** ***N*** ** = 3,149)** ***n*** **(%)**	**No rivaroxaban + no ASA** **(** ***N*** ** = 2,948)** ***n*** **(%)**	***p*** **-Value (rivaroxaban + ASA vs no rivaroxaban + no ASA)**
Major Bleeding	8 (0.28)	6 (0.20)	9 (0.29)	3 (0.10)	0.11
NMCR Bleeding	38 (1.34)	28 (0.92)	47 (1.49)	23 (0.78)	0.009

Abbreviations: ACM, all-cause mortality; ASA, Aspirin; CV, cardiovascular; ITT, intention to treat; MI, myocardial infarction; NMCR, non-major clinically relevant, Sx, symptomatic; VTE, venous thromboembolism.

As a sensitivity analysis, we adjusted for differences in baseline demographic covariates. The final model included age, D-dimer, and baseline diastolic BP but treatment was no longer significant ( *p* = 0.063). However, when an adjusted model was used for symptomatic VTE or ACM, treatment with R/A remained significant compared with no TP (hazard ratio [HR], 0.60; 95% confidence interval [CI], 0.40–0.90; *p* = 0.014) as it did in an adjusted model for ACM alone (HR, 0.63; 95% CI, 0.41–0.98; *p* = 0.041).

MB was low in all groups but numerically higher in the groups with rivaroxaban (0.28 and 0.29% with rivaroxaban alone and R/A, respectively), intermediate with aspirin alone (0.20%), and lowest in the no TP group (0.10%). NMCR bleeding was increased with combination therapy compared with no TP (1.49 vs 0.78%, *p* = 0.009).

## Discussion

In this post hoc analysis of the MARINER trial among patients who were at elevated risk of VTE in the immediate postdischarge period, the use of rivaroxaban plus aspirin at baseline was associated with a significant 40% lower risk of symptomatic VTE and VTE-related death, and a significant 52% lower risk of symptomatic VTE and ACM compared with no TP. Patients receiving dual antithrombotic therapy also experienced a 43% lower risk of ACM compared with those receiving no treatment. Moreover, dual antithrombotic therapy showed an advantage over monotherapy with either rivaroxaban or aspirin alone without numerically important increases in MB, despite increased NMCR bleeding.

Previous studies of postdischarge thromboprophylaxis with DOACs have shown significant reductions in VTE among subgroups of patients with specific risk factors, particularly age over 75 years, elevated D-dimer, and IMPROVE score ≥ 4. 7 8 15 Additional analyses of DOACs in these cohorts have shown reductions in major and fatal CV events. 13 16 17 Substudies of the APEX trial have shown that extended thromboprophylaxis with betrixaban reduces ACM, stroke, and ischemic stroke, particularly among high-risk patients with ischemic stroke or congestive heart failure as the index event, 16 as well as fatal or irreversible events, particularly among patients with elevated D-dimer, 13 compared with standard prophylaxis. A prespecified MARINER subanalysis showed a 28% reduction in major and fatal thromboembolic events without increased MB among patients randomized to rivaroxaban 10 mg daily compared with placebo. 17

The potential advantage of antithrombotic therapy with dual pathway inhibition in reducing major thromboembolism and mortality in our cohort is suggested by results of the COMPASS trial, which showed a 24% relative risk reduction in the composite of stroke, MI, and CV death with low-dose rivaroxaban (2.5 mg twice daily) in combination with low-dose aspirin, compared with low-dose aspirin alone, in individuals with chronic CV disease over a mean follow-up period of 23 months. 11 This regimen in COMPASS suggested reductions in VTE in these patients as well. The increased MB among COMPASS patients receiving long-term dual therapy was, not surprisingly, an order of magnitude higher than in the present study (3.1 vs 0.29%), which evaluated dual therapy only up to 45 days postdischarge. Significantly lower incidences of symptomatic VTE/VTE-related death as well as ACM, with low absolute incidence of MB, suggest a favorable benefit/risk profile for patients who receive dual pathway inhibition with low-dose anticoagulants plus aspirin during the immediate period following acute medical illness.

Since aspirin use was not randomized, comparison of efficacy outcomes can only give an estimate of effectiveness. For example, the incidence of symptomatic VTE or VTE-related death was similar in the rivaroxaban-alone group (0.91%) compared with the aspirin-alone group (0.92%). The comparison of all rivaroxaban versus all placebo, which was randomized, was previously reported (0.83 vs 1.10%). 8 Interestingly, the comparison of all aspirin versus all no aspirin, which was not randomized, yields similar results (0.84 vs 1.10%, respectively), but may certainly be confounded by indication for aspirin use. Nevertheless, the same dose of rivaroxaban (10 mg daily) was compared with low-dose aspirin (81 mg) in a large, randomized study evaluating symptomatic VTE after hip or knee arthroplasty (after an initial 5-day course of rivaroxaban in both groups), and both regimens were found to be similar in preventing symptomatic VTE (0.70 vs 0.64%, respectively). 9 Our hypothesis for this study was that dual antithrombotic therapy (rivaroxaban and low-dose aspirin) would be superior to no antithrombotic therapy.

In an attempt to adjust to differences in covariates between groups at baseline, a Cox proportional hazards model was created using a backward selection process including treatment (R/A vs no TP). When adjusting for baseline demographic covariates associated with the primary end point, significance was lost for the treatment comparison ( *p* = 0.063). However, this was not the case in adjusted models for symptomatic VTE and ACM ( *p* = 0.014) or ACM alone ( *p* = 0.041). These results support the notion that the combination of rivaroxaban and aspirin may reduce important outcomes compared with no treatment for medically ill patients after discharge.

The present study has several strengths, including the large population of at-risk individuals and central adjudication of outcomes in the original study. Results should be interpreted cautiously, however, as the present analyses were not prespecified, aspirin use at baseline was not randomized, and aspirin use beyond baseline was not recorded. Therefore, only effectiveness can be assessed, and the results may be confounded as aspirin use is clearly higher in certain population (e.g., patients with ischemic stroke and heart failure). The adjusted analyses partially support effectiveness, but not on the primary end point and there was no adjustment for multiplicity. In addition, other confounders could still exist that were not captured in the model. Finally, the number of patients with MB was very small and thus comparisons may be underpowered to detect clinically meaningful differences. Notwithstanding these limitations, this study adds to existing literature suggesting an advantage of dual pathway inhibition as an antithrombotic strategy in reducing major and fatal thrombotic events among cohorts of high-risk medically ill individuals.

## Conclusion

Extended postdischarge thromboprophylaxis with rivaroxaban in addition to baseline aspirin use was associated with fewer thromboembolic events and a lower rate of VTE-related death compared with lack of either therapy in previously hospitalized medical patients at risk for major thrombotic events in an unadjusted analysis. This observation lost significance after adjustment for differences in covariates at baseline. However, even after adjustment, there were nominally significant reductions in symptomatic VTE and ACM as well as ACM alone. Efficacy results were intermediate and similar in the rivaroxaban-alone or aspirin-alone groups. The incidence of MB was similar among groups, though NMCR bleeding was increased with dual antithrombotic therapy compared with no TP. These findings suggest the need for confirmation in a prospective, randomized trial.

## Essentials

- A DOAC plus aspirin may reduce postdischarge thrombosis compared with no antithrombotic therapy.
- This post hoc analysis of the MARINER trial compared rivaroxaban, aspirin, both, or neither as postdischarge thromboprophylaxis.
- Dual antithrombotic therapy was associated with fewer VTE events and death than no prophylaxis.
- Considering low absolute bleeding rates, dual antithrombotic prophylaxis merits study in prospective trials.

## Notes

Appendix A Submission of a suspected event for adjudication by the Clinical Events Committee (who was blinded to treatment assignment) was required for:

- All deaths.
- Suspected events that included symptoms or signs suggestive of PE or DVT.
- Performance of any imaging or other diagnostic study that could have yielded an incidental diagnosis of PE or DVT.
- Suspected events that included acute coronary syndrome or myocardial infarction. Chest pain determined not to be cardiac in origin did not need to be sent for adjudication.
- Suspected events that included symptoms compatible with a transient ischemic attack or stroke. Events determined not to be a transient ischemic attack or stroke (e.g., seizure, hypoglycemia) did not need to be sent for adjudication.
- Any study outcome event (efficacy or safety) found on autopsy was reported on the appropriate outcome event page in the clinical database.

Any clinical event that suggested the possibility that an efficacy outcome event had occurred (including acute coronary syndrome and transient ischemic attack) was reported on the appropriate case report form page and sent for adjudication. Ultrasound imaging studies from cases of suspected DVT were evaluated. Adjudicated results were used for the final analyses. All deaths were adjudicated as far as the cause of death by the Clinical Events Committee using all available information, including but not limited to a specific death case report form page, a hospital admission history and physical, a death summary, and an autopsy report.
